# Brain Health Services: organization, structure, and challenges for implementation. A user manual for Brain Health Services—part 1 of 6

**DOI:** 10.1186/s13195-021-00827-2

**Published:** 2021-10-11

**Authors:** Daniele Altomare, José Luis Molinuevo, Craig Ritchie, Federica Ribaldi, Emmanuel Carrera, Bruno Dubois, Frank Jessen, Laura McWhirter, Philip Scheltens, Wiesje M. van der Flier, Bruno Vellas, Jean-François Démonet, Giovanni B. Frisoni, on behalf of the European Task Force for Brain Health Services, Marc Abramowicz, Daniele Altomare, Frederik Barkhof, Marcelo Berthier, Melanie Bieler, Kaj Blennow, Carol Brayne, Andrea Brioschi, Emmanuel Carrera, Gael Chételat, Chantal Csajka, Jean-François Demonet, Alessandra Dodich, Bruno Dubois, Giovanni B. Frisoni, Valentina Garibotto, Jean Georges, Samia Hurst, Frank Jessen, Miia Kivipelto, David Llewellyn, Laura McWhirter, Richard Milne, Carolina Minguillón, Carlo Miniussi, José Luis Molinuevo, Peter M. Nilsson, Janice Ranson, Federica Ribaldi, Craig Ritchie, Philip Scheltens, Alina Solomon, Wiesje van Der Flier, Cornelia van Duijn, Bruno Vellas, Leonie Visser

**Affiliations:** 1 Laboratory of Neuroimaging of Aging (LANVIE), University of Geneva, Geneva, Switzerland; 2 Memory Clinic, Geneva University Hospitals, Rue Gabrielle-Perret-Gentil 6, 1205 Geneva, Switzerland; 3 Barcelonaβeta Brain Research Center, Pasqual Maragall Foundation, Barcelona, Spain; 4 Centre for Clinical Brain Sciences, University of Edinburgh, Edinburgh, UK; 5 Laboratory of Alzheimer’s Neuroimaging and Epidemiology (LANE), Saint John of God Clinical Research Centre, Brescia, Italy; 6 Department of Molecular and Translational Medicine, University of Brescia, Brescia, Italy; 7 Department of Neurology, Stroke Center, University Hospitals and University of Geneva, Geneva, Switzerland; 8 Institut de la Mémoire et de la Maladie d’Alzheimer, IM2A, INSERM, Institut du Cerveau et de la Moelle Épinière, UMR-S975, Groupe Hospitalier Pitié-Salpêtrière, AP-HP, Sorbonne Université, Paris, France; 9 Department of Psychiatry and Psychotherapy, Medical Faculty, University of Cologne, Cologne, Germany; 10 Department of Neurology, Alzheimer Center Amsterdam, Amsterdam Neuroscience, Vrije Universiteit Amsterdam, Amsterdam UMC, Amsterdam, The Netherlands; 11 Life Science Partners, Amsterdam, The Netherlands; 12 Department of Epidemiology and Biostatistics, Vrije Universiteit Amsterdam, Amsterdam UMC, Amsterdam, The Netherlands; 13 Gérontopole of Toulouse, University Hospital of Toulouse (CHU-Toulouse), Toulouse, France; 14 Centre Leenaards de la Mémoire, Centre Hospitalier Universitaire Vaudois, Lausanne, Switzerland

**Keywords:** Brain Health Services, Dementia, Aging, Alzheimer’s disease, Prevention, Dementia risk, Risk communication, Risk reduction, Cognitive enhancement, Personalized medicine

## Abstract

Dementia has a devastating impact on the quality of life of patients and families and comes with a huge cost to society. Dementia prevention is considered a public health priority by the World Health Organization. Delaying the onset of dementia by treating associated risk factors will bring huge individual and societal benefit. Empirical evidence suggests that, in higher-income countries, dementia incidence is decreasing as a result of healthier lifestyles. This observation supports the notion that preventing dementia is possible and that a certain degree of prevention is already in action. Further reduction of dementia incidence through deliberate prevention plans is needed to counteract its growing prevalence due to increasing life expectancy.

An increasing number of individuals with normal cognitive performance seek help in the current memory clinics asking an evaluation of their dementia risk, preventive interventions, or interventions to ameliorate their cognitive performance. Consistent evidence suggests that some of these individuals are indeed at increased risk of dementia. This new health demand asks for a shift of target population, from patients with cognitive impairment to worried but cognitively unimpaired individuals. However, current memory clinics do not have the programs and protocols in place to deal with this new population.

We envision the development of new services, henceforth called Brain Health Services, devoted to respond to demands from cognitively unimpaired individuals concerned about their risk of dementia. The missions of Brain Health Services will be (i) dementia risk profiling, (ii) dementia risk communication, (iii) dementia risk reduction, and (iv) cognitive enhancement. In this paper, we present the organizational and structural challenges associated with the set-up of Brain Health Services.

## Background

Dementia consists of the cognitive decline from a previous level of performance to such an extent that it interferes with independence in everyday activities [1]. It impacts patients and their families and comes with a huge cost to society. Dementia prevention therefore is considered a public health priority by the World Health Organization [2]. Delaying the onset of dementia by treating underlying diseases will bring huge individual and societal benefit. Empirical observations in cohorts born in more recent decades in high-income countries indicate a reduction of the age-specific incidence of dementia [3–14], suggesting that dementia prevention is possible and already in action. This is likely the unintended result of greater overall wealth and healthier lifestyles, including better control of cardiovascular risk factors. Epidemiological evidence allows to estimate that 40% of dementia cases are due to lifestyle and cardiovascular modifiable risk factors [15], while the remaining cases are largely explained by genetic (e.g., *APOE* ɛ4), biological (e.g., amyloid and tau), other unknown risk factors, and their interactions [16]. However, the bad news is that dementia prevalence is steadily increasing worldwide. This is due mainly to population aging in lower- and middle-income countries and to the increased life span of individuals with dementia in higher-income countries. Therefore, a further decrease of dementia incidence is needed to counteract the worldwide trend of increased dementia prevalence. We believe that, today, evidence is sufficient to set up evidence-based, effective, personalized, and equitable dementia prevention plans in persons at risk of dementia.

The current memory clinics have been designed for the needs of patients with overt cognitive and/or behavioral disorders with the aim of reducing the burden of progressive decline (*tertiary prevention*). Nevertheless, a considerable number of cognitively unimpaired individuals believing that they may be at increased risk of dementia is seeking help in memory clinics, accounting for 20–30% of all patients [17–19]. Increasing and consistent evidence indicates that these have a mildly increased risk of dementia as compared to the general population [20]. These individuals present specific concerns, requests, expectations, and hopes different from those of the cognitively impaired ones, but they are usually discharged with generic recommendations and reassurance, and no really actionable and meaningful answers.

The development of new and innovative services, henceforth referred to as Brain Health Services (BHSs), is needed to provide specific answers to these individuals’ unmet needs. The missions of BHSs consist of (i) dementia risk profiling, (ii) dementia risk communication, (iii) dementia risk reduction (*primary* and *secondary prevention*), and (iv) cognitive enhancement. BHSs will feature specific knowledge, skills, protocols, and technology to meet the challenges posed by this new demand. Some pilot experiences are ongoing at the time of the writing of this article (Q4 2020) in Barcelona, Edinburgh, and Paris and have provided ideas and tools for this article and the others of this series published in this issue of *Alzheimer’s Research & Therapy*.

This is the first of six papers, which are part of a larger initiative of the European Task Force for Brain Health Services, aiming to draft the protocols of operations in the BHSs of the future. Here, we describe the organization, structure, and challenges for implementing BHSs, while the other papers focus on the four missions (mentioned above) and on the societal challenges.

## BHS organization

In this section, we present how the novel BHS facilities might be structured at the time of writing of this article (Q4 2020). In Section 4.1, we envision how BHSs may look like in the upcoming years based on research advances and technological innovations.

### What is in a name?

Equally tenable denominations could be proposed, emphasizing different aspects: i) the biomedical domains (e.g., brain, dementia, Alzheimer’s disease, memory), (ii) the clinical mission (e.g., health, prevention), and (iii) the organizational structure (e.g., clinic, service, unit). The end result would be labels such as “brain health clinics,” “dementia prevention services,” etc.

We propose “Brain Health Services” for the following reasons: (i) the concept of “brain health” is more comprehensive than “dementia prevention,” opening to cognitive enhancement which, by definition, aims to improve cognitive functions rather than preventing dementia, and is one of people’s demands and (ii) the terms “clinic” and “unit” imply that the services would be delivered in structures independent of other services, while we believe that BHSs can be implemented either within the current memory clinics or as distributed and interconnected services (see the “Context for BHS implementation” section). Whatever the label, its semantic should match the content of the health offer.

### Users

The target population of BHSs will consist of older or middle-aged adults who wish to check their risk of dementia, preserve cognitive functions, or enhance their cognitive performance. This population includes individuals with subjective cognitive decline (SCD) [21], functional cognitive disorders [19], and the “worried wells.”

Individuals with SCD experience persistent cognitive decline which is not detected by the standard clinical and neuropsychological batteries used to detect mild cognitive impairment and dementia and, although cognitively unimpaired, have an increased risk of dementia as compared to the general population (incidence of 20.1/1000 person-year vs 14.2/1000 person-year [20]). Functional cognitive disorders consist of a range of overlapping conditions in which cognitive symptoms, usually of attentional nature, present characteristic internal inconsistency as the result of reversible changes in brain function rather than damage or disease [19]. Functional cognitive disorders may present as an isolated syndrome, or in the context of anxiety or depression, or alongside other functional or somatoform symptoms such as chronic pain [19]. Some individuals with functional cognitive disorders may perform in the mildly impaired range on cognitive tests [19]. Where a positive diagnosis of functional cognitive disorders is made, appropriate treatment should include a clear explanation of the diagnosis using supportive written material (for example, “Functional Neurological Disorder (FND): a patient’s guide” [22]). Worried wells do not have any specific cognitive complaint, but they claim concern of declining cognition in the future, and strive to preserve it as long as possible or even enhance it. Worried wells frequently report a family history of dementia or Alzheimer’s disease.

Clearly, the target population of BHSs is remarkably different from that of current memory clinics. The above case-mix suggests not to refer to them as “patients,” but to rather prefer a more neutral term such as “users.”

It is worth noting that the definition and identification of the ideal target population might not always be clear-cut, especially when it comes to individuals with borderline or inconclusive cognitive testing or very mild cognitive or executive dysfunctions who might still benefit of the BHS offers. Moreover, while we expect that a larger share of users might be classified as SCD, functional cognitive disorders or worried wells, other groups might access BHSs. These groups might include individuals with mild forms of mood, anxiety, sleep, attention-deficit/hyperactivity, or other disorders. Some of these individuals might be eligible to enter a BHS journey depending on BHS personnel and facilities (e.g., a psychiatrist or psychotherapist with experience in mood disorders), while others should be referred to external specialists. Individuals with severe psychiatric or physical comorbidity (e.g., cardiovascular/cerebrovascular diseases) will not represent the target population of BHSs.

In the perspective of growing demand and growing offer, in the early years of BHSs, users will be referred by memory clinics. At a later stage, as BHSs catch up, they will consolidate their own user flow consisting of people who spontaneously show up directly to BHSs. The implementation of educational programs (e.g., awareness campaigns on brain health for the general population) and other initiatives [23] might increase the BHSs visibility and reputation.

### Missions

The four main missions of BHS are as follows: (i) dementia risk profiling, (ii) dementia risk communication, (iii) dementia risk reduction (*primary and secondary prevention*), and (iv) cognitive enhancement. These topics are exhaustively discussed in the pertinent papers [24–27], and briefly summarized below.

Education of the general public and health care providers might be a mission of BHSs in academic settings. This will not be addressed in this paper as it will be of interest to a minority of academic BHSs and is beyond the scope of this initiative.

#### Dementia risk profiling

The very first step of assessment in BHSs will be understanding the user’s request. Anecdotal observations indicate that a number of individuals with SCD, functional cognitive disorders, or worried wells look for reassurance. Indeed, malaises such as psychological/psychiatric (e.g., depression, trauma, affective issues) or personal issues (e.g., divorce, violent spouse, unemployment, societal issues) are sometimes presented in disguise as “memory” concerns. A careful history collection, carried out with tact and empathy, can be revealing. In such cases, a “blind” offer of dementia risk assessment would be a clinical misstep. The BHS clinician should here refer the user to the appropriate specialist.

The following step to the implementation of personalized prevention plans is to identify users’ risk factors for dementia. The relative risk of modifiable dementia risk factors varies widely between 1.1 for air pollution to 1.9 for hearing loss and depression [15] and dramatically increases for genetic (*APOE* ɛ4 genotype) and biological (amyloid and tau deposition) risk factors [16]. BHSs must be able to comprehensively assess, combine (e.g., through composite dementia risk scores such as the Cardiovascular Risk Factors, Aging, and Incidence of Dementia (CAIDE) Dementia Risk Score [28]; the Brief Dementia Screening Indicator [29]; and the Australian National University Alzheimer’s Disease Risk Index [30, 31]), and interpret all these risk factors together with protective factors, and to finally profile and categorize the user’s specific risk into strata (e.g., high, moderate, or low risk of developing dementia in the following 5, 10, or 15 years). A basic level of assessment should include sociodemographic, lifestyle, and health risk factors, followed by *APOE* status and biomarkers if resources allow. Importantly, risk profiling should take into account other demographic variables such as age, gender, and ethnicity which might influence the interpretation of risk factors (e.g., *APOE* ɛ4 is associated with a higher risk of AD in individuals aged 65–70 years [32], in women as compared to men [33, 34], and in Japanese and Caucasian individuals as compared to African Americans and Hispanics [35]).

Further research is still needed to estimate the relative risk of each risk factor adjusted for communality with other factors; develop composite risk scores combining modifiable, biological (e.g., amyloid and tau), and genetic (e.g., *APOE*) risk factors; and develop cost-effective screening protocols [24].

#### Dementia risk communication

We recommend disclosing the risk of dementia, whether based only on lifestyles or also on genetic or biomarker status, to users, providing expert counseling if necessary. However, this decision should be taken on an individual basis and taking into account user’s cultural, societal, and economic backgrounds, belief system, and expectations.

The communication of the concept of risk to the general public is challenging, especially in the context of untreatable and stigmatized neurodegenerative diseases leading to dementia. Evidence on how to communicate dementia risk is scarce. Nevertheless, the available literature allows to put in place some practical recommendation [25]. These recommendations are inspired from other fields with more experience on this topic (e.g., oncology [36–38]) and from existing research disclosure protocols of genetic (e.g., *APOE* genotype [39–42]) and biomarker (e.g., amyloid-PET [43–47]) results that proved to have a well-tolerated psychological impact in the short term. Nevertheless, we acknowledge that these disclosure protocols are limited to explaining that genes or biomarkers are risk factors for dementia, but do not actually communicate the risk of developing dementia in the next few years.

We underline that the use of standardized communication protocols on an individual level is challenging and might require a certain degree of adaptation and clinical sensitivity. Further research is needed to develop communication protocols delivering quantitative information about individual risk, and scalable tools suitable to users with different socio-demographic and cultural features (including educational background). BHSs will represent the ideal context for this research.

Finally, BHSs will need to deal with the ethics regulations and potential regulatory hurdles associated with the communication of risk (mainly so if based on genetics) to the users, their families, and other actors (e.g., healthcare insurances, employers). Informed consent should be given before entering a BHS journey, and confidentiality should be enforced at all levels of the BHS journey.

#### Dementia risk reduction

Risk reduction interventions aim to reduce the likelihood of long-term cognitive decline or dementia onset in at-risk individuals. Among all the randomized trials on multi-domain interventions, only the FINGER study met its primary outcome, showing greater cognitive improvement in participants of the experimental group versus controls [48]. On the contrary, other randomized trials on multi-domain interventions such as MAPT [49], preDIVA [50], Look AHEAD [51], and DO-HEALTH [52] failed to meet their primary outcomes.

Interestingly, subsample analyses of the FINGER and MAPT studies showed that interventions were more effective in patients at increased risk for dementia based on genetic (*APOE* ɛ4) [53] or biological (amyloid positivity) [49] risk factors. This suggests that personalized multi-domain interventions, tailored to the individual’s specific risk factors (reflecting the risk reduction potential), are likely associated to the highest clinical benefit.

Even though preliminary evidence is now available and allows to provide recommendations for practical implementation of precision dementia risk reduction interventions [26], long-term multi-domain randomized controlled trials are needed to provide definitive evidence on their efficacy. The World Wide-FINGERS, the first network for multimodal dementia prevention trials, aims to fill this evidence gap by adapting and optimizing the FINGER operational model for dementia risk reduction in different populations, and geographic and economic settings [54].

Translation of experimental risk reduction interventions to the clinical setting will not be straightforward. Possible interventions that can be offered to BHS users today or in the next few years might cover one of more of the following areas: diet, exercise, cognitive training, and vascular risk monitoring (inspired by current cardiovascular prevention programs) [26].

Finally, we underline the importance of correcting sensory impairment or emotional disorders (e.g., anxiety or depression) as a prerequisite for the implementation of effective dementia risk reduction intervention.

#### Cognitive enhancement

Cognitive enhancement interventions aim to improve the individual’s performance and abilities. These interventions are typically performed over a time span of a few days/weeks. Cognitive enhancement interventions include cognitive, mental, and physical training (including mindfulness); non-invasive brain stimulation; and cognitive-enhancement drugs. To date, currently available evidence supporting the efficacy of cognitive training is limited and heterogeneous but generally positive, while that supporting the efficacy of mindfulness and tDCS interventions might possibly increase in the next few years. Evidence on cognitive-enhancing drugs is poor and inconclusive [27].

### Personnel and expertise

The dementia domain is largely interdisciplinary and spans neurology, geriatrics, psychiatry, cognitive psychology, neuropsychology, nursing, and social sciences. Indeed, BHSs should be led by multidisciplinary teams to cover all these areas. Expertise in psychology and/or neuropsychology is necessary for the initial (and potential follow-up) clinical and cognitive evaluations, to communicate the risk, and to implement cognitive interventions. Medical expertise (e.g., in neurology, geriatrics, psychiatry) is necessary to define indications for entering the BHS track, carry out exams, interpret biological and genetic risk factors, prioritize risk, set risk reduction interventions, and propose follow-up if needed. Nursing competences might be necessary to collect samples and measures for risk factor assessment (e.g., biological samples, blood pressure). Further expertise, such as nutrition or a physical training, might be useful to cover some specific areas of prevention.

BHSs will wish to recruit personnel based on the required expertise rather than on a priori defined professional categories. For example, although current job descriptions usually suggest that dementia risk communication should be done by a physician, we believe that a psychologist with appropriate training, empathy, and communication skills can safely perform this task. Post-graduate courses on the care of persons with cognitive disorders that are active or being launched in Europe will help educate BHS professionals [55].

### Basic vs advanced BHSs

Not all BHSs will need to cover the whole range of potential health offer. We envision at least two levels, basic and advanced, depending on resources and available facilities. Basic services may consist of (i) standardized risk assessment based on lifestyles, vascular and basic genetic risk factors (e.g., *APOE*), and possibly measures reflecting structural brain health (e.g., qualitative or quantitative measures of atrophy and vascular changes), implementing low-level composite dementia risk scores (e.g., the CAIDE Dementia Risk Score); (ii) adaptation and use of current practices for dementia risk communication; (iii) implementation of standardized non-pharmacological *primary prevention* protocols (e.g., FINGER and MAPT interventions) and pharmacological and non-pharmacological control of cardiovascular risk factors; and (iv) cognitive enhancement using cognitive training.

An advanced version of BHSs may expand the basic services with one or more of the following: (i) molecular imaging biomarkers (e.g., amyloid-PET, tau-PET, MRI with automated image post-processing) and/or CSF biomarkers (e.g., Aβ_42_, phosphorylated tau, neurofilament light), (ii) use of structured personalized dementia risk communication protocols taking into account user’s specific features (e.g., educational background), (iii) implementation of personalized *primary* and *secondary prevention* protocols tailored to the user’s molecular risk profile including biomarker derived information, and (iv) combination of sophisticated and personalized cognitive enhancement techniques (e.g., cognitive training and non-invasive brain stimulation).

As of today, some of basic BHSs’ activities (e.g., *primary prevention*) could be absorbed by general practice depending on the structure of local healthcare provision and local opportunities. On the contrary, at the current state of science and technology, most of the advanced BHSs’ activities (e.g., *secondary prevention*) cannot take place in the general practice. The availability of blood-based biomarkers may change the scenario only if shown to be sufficiently specific and in the presence of a well-tolerated preventive drug.

### Facilities

The main technological facilities needed in BHSs are largely the same of a traditional memory clinic and might include MRI, PET, and fully automated CSF analysis platforms (e.g., Elecsys, Lumipulse).

Other facilities will be specific to BHSs and may include tablets for computerized cognitive training, physical activity monitors, and fitness trackers.

As is the case of current memory clinics, local factors such as availability of technology or expertise, or idiosyncrasies towards a given diagnostic or intervention technology will give individual BHSs their specificity.

### Context for BHS implementation

BHSs can be either hybrid or stand-alone services. In the first case, BHSs can leverage on the current memory clinics’ structure and ongoing collaborations (with nuclear medicine, radiology, biochemistry laboratories, etc.). BHS-specific expertise and technology will need to be integrated, since some personnel and facilities are often lacking in memory clinics such as psychotherapists, nutrition experts, physical trainers, and devices for transcranial stimulation. The investment in this case would be relatively modest. In the second case, stand-alone BHSs will need new personnel and facilities and to build collaborations with other services. The investment in this case would obviously be significantly higher. In either case, since stroke centers are already dealing with the implementation of cardiovascular prevention programs and the promotion of awareness-rising campaigns (both key aspects of BHSs), BHSs can partner with them and leverage on their longstanding expertise.

Whatever the setup, a tight collaboration between BHSs and memory clinics is strongly encouraged by this working group. Indeed, memory clinics can refer cognitively unimpaired patients to BHSs in order to investigate their request and provide meaningful answers. Conversely, BHSs can refer cognitively impaired users to memory clinics in order to start proper diagnostic workup and treatment.

### Similar initiatives

The BHS initiative has some similarities with previous initiatives. For example, a similar approach has been previously adopted by two Alzheimer’s Prevention Clinics whose mission is to provide personalized therapeutic interventions, based on the individual risk profile, in patients at risk for AD [56]. This approach was clearly presented in a paper describing in detail the supporting methodology as well as the proposed risk reduction interventions and the associated challenges [56]. Briefly, patients entering the Alzheimer’s Prevention Clinics undergo a basic assessment of genetic, lifestyle, and cardiovascular risk factors followed by personalized therapeutic interventions. We acknowledge that the basic BHSs (as described in the “Basic vs advanced BHSs” section) might resemble, at least in some aspects, the Alzheimer’s Prevention Clinics. Nevertheless, the advanced BHSs will allow to take a step forward towards a more comprehensive and accurate risk profiling by assessing molecular biomarkers, and more effective risk reduction interventions tailored to the user’s molecular risk profile.

In the last few years, several “trial-ready” cohort projects have been launched, the most relevant of which are EPAD [57] and TRC-PAD [58]. Such projects aim to recruit and screen participants in order to assemble deeply phenotyped cohorts which provide a pool of participants to clinical trials. Indeed, the only offer of “trial-ready” projects is inclusion in clinical trial, and their target population most of the times consists only of amyloid-positive individuals. Differently, BHSs will deliver multiple pharmaceutical and non-pharmaceutical risk reduction interventions and their target population in broader covering older or middle-aged adults with variable risk profiles. These differences make the BHS initiative unique of its kind.

## BHS challenges

### Equity and societal challenges

One of the main challenges will consist in making BHSs equitable, i.e., accessible to the general population regardless of their economic status. Most interventions potentially offered by BHSs are not reimbursed in any Western country; they may take place in for-profit enterprises where users pay interventions with out-of-pocket money. Indeed, BHSs, at least at their first development stages, will thrive mainly in higher-income countries for the greater social awareness of cognitive diseases. As a consequence, access may be limited to the more affluent and more highly educated members of society. Paradoxically, the population that would benefit the most from the BHSs is the one likely to be excluded (at least initially). Indeed, individuals with disadvantaged conditions, lower education, and lower socioeconomic status are likely those with higher risk of dementia and who might benefit the most from risk reduction and cognitive enhancement interventions. See Milne et al. [59] for a deeper discussion on this topic. The affiliation to an existing memory clinic or stroke center might facilitate the coverage at least some procedures by healthcare insurances.

### Individual interventions vs large-scale population interventions

The European Task Force for Brain Health Services is largely made of clinicians and clinical researchers who are by mission focused on individuals rather than on society as a whole. Indeed, even though BHSs can sporadically touch the general population (e.g., by awareness promoting campaigns on brain health), their mission is the implementation of personalized prevention plans tailored to the individual’s risk profile. This is the so-called “high-risk approach” that has contributed to dramatically decrease stroke morbidity and mortality over the past decades [60].

Nevertheless, the authors acknowledge that well-designed and implemented prevention initiatives at the population level might be associated with great societal benefit, if only in the long term. Such interventions require the direct engagement of healthcare systems and payers and strong evidence supporting the efficacy of interventions [61]. BHSs may contribute to the production of this evidence, while they may or may not be the hubs of prevention initiatives at the population level.

### Sustainability

Depending on the context (see the “Context for BHS implementation” section), a BHSs will require variable amount of funding to be financially sustainable. It is likely that business models for BHSs will develop through several stages. Initial resources may come from grants, philanthropy, and channeling research income/overheads into the establishment of innovator sites that will by necessity be located in university teaching hospitals. Such settings will not need to invest heavily in up-front capital costs for, e.g., MRI scanners. These settings must commit to generating substantial evidence on access and health outcomes to deliver both short- and long- term health economic analysis. These will be locally derived to take to the local health care funders and will be nuanced to reflect the needs/motivations of the purchaser.

The purchasing by the extant health providers has to be the exit strategy for the reactive initial funding. One could argue that a 5-year period of funding for “pilot or innovator” sites is sufficient to make the argument to transition to centralized funding by, e.g., Healthcare Commissioners in the NHS. This will be supported by, e.g., NICE guidance and other clinical policy documents that will support individual practitioners in making their business case. Reports from advocacy groups whilst helpful are no replacement for policy documents generated in an unbiased fashion by organizations like NICE. Finally, the patient perspectives on the service can act as a powerful motivator for change. Collecting data on their experience will help the development of services as well as their extension to other regions of the country in question.

Of course, investors in the market of private healthcare may also wish to seize the opportunity of investing in this growing market. The setups of BHSs in already existing structures (e.g., memory clinics or stroke centers) will minimize the amount of the investment.

### Research

In order to promote equity and sustainability, BHSs should integrate their offer with continuing research activity. Sound evidence produced by BHSs research activity might contribute to (i) identifying the trajectories of the underlying pathologies by the follow-up of individuals at a preclinical stage, (ii) selecting individuals at high risk for the inclusion in clinical trials aimed at studying the efficacy of disease-modifying therapies at a preclinical asymptomatic stage of the disease, (iii) producing strong scientific evidence on the efficacy of interventions (or lack thereof), (iv) making structural efforts to access more marginalized communities by design, and (v) drawing attention of healthcare systems and persuade them to provide coverage, making BHS sustainable and equitable.

## Discussion

The increasing prevalence of dementia, the awareness of the general population on brain health, recent advancements in technology and knowledge of neurodegenerative diseases, and preliminary evidence of effective risk reduction interventions constitute the rationale behind the development of BHSs. BHSs will focus on a new target population (cognitively unimpaired individuals concerned with the preservation or improvement of their cognitive abilities); have specific missions (dementia risk profiling, dementia risk communication, dementia risk reduction, and cognitive enhancement); face relevant challenges (demonstrating efficacy, equity and sustainability of the services); and require high-level expertise, facilities, and personnel. BHSs might rely on the current memory clinics or be independent services.

The current aim of this BHS initiative is to raise awareness on the need for new services aimed at currently underserved group individuals and provide a large set of recommended interventions which should be locally adapted by healthcare providers based on local needs and resources.

### The future of BHSs

We envision that BHSs might change in the upcoming years thanks to research advances and novel technologies.

#### Dementia risk profiling

The clinical validation of blood-based biomarkers of amyloid [62], tau [63], and neurodegeneration (e.g., neurofilament light [64]) will radically change the way individual risk is assessed. Indeed, blood-based biomarkers are much cheaper than molecular imaging and much more accessible. We envision a scenario where blood-based biomarkers with high sensitivity for abnormality will be used for large-scale dementia screening, thus reducing the number of users requiring more expensive testing. Polygenic risk scores may also make the transition to clinical fruition in the coming years. The widespread use of calculators (e.g., ADappt [65]) will allow a comprehensive interpretation of multiple risk factors and the quantification of the user’s risk. Finally, the use of brain health registries [66, 67] and digital tools will facilitate the access of users to BHSs.

#### Dementia risk communication

Large-scale education programs will result in increased awareness of the general population on brain health. A more educated and aware population has a better predisposition to understand the concept of risk. Nevertheless, further research is needed to develop and implement proper communication strategies on an individual level.

#### Dementia risk reduction

Aducanumab [68, 69] might be the very first disease-modifying therapy approved by the FDA for clinical use in patients with prodromal Alzheimer’s disease or mild Alzheimer’s disease dementia. Several phase 3 clinical trials on anti-amyloid drugs in cognitively healthy individuals are currently ongoing, and their results are expected between 2021 and 2025 [70]. If they prove to be effective, disease-modifying therapies will be the main weapon to prevent cognitive deterioration in cognitively unimpaired biomarker-positive individuals. In the optimistic scenario of an effective disease-modifying therapy, BHSs will play a key role in screening the population and delivering such therapies.

However, whether disease-modifying therapies will be available or not, more targeted personalized multidomain interventions will be increasingly fine-tuned and implemented in BHSs [54].

#### Cognitive enhancement

In the next few years, protocols combining cognitive training, mindfulness, and non-invasive brain stimulation might be available, although the timelines are even harder to predict than for industry-sponsored pharmacological clinical trials.

This provides an example of how BHSs might operate when blood-based biomarkers are available. In this scenario, users undergo screening including assessment of *APOE* genotype and lifestyle risk factors as well as high-sensitivity blood-based biomarkers. Those users with positive or borderline blood-based biomarkers might also undergo a second-level assessment with molecular imaging (e.g., amyloid-PET, tau-PET), even if this might be not necessary if blood-based biomarkers prove to be highly accurate (in terms of both high sensitivity and specificity). Taken together, this information allows to profile the user’s risk and classify it (e.g., as “low,” “moderate,” or “high” based on a composite relative risk). Afterward, the risk is communicated to the user. Finally, the intervention is chosen accordingly: users with low risk might start personalized cognitive enhancement interventions, while users with moderate or high risk should undergo personalized risk reduction interventions possibly including disease-modifying therapies. This is a purely indicative scenario and can vary based on the context of BHS implementation. CT, computerized tomography; MRI, magnetic resonance imaging; FDG, fluorodeoxyglucose; PET, positron emission tomography; *APOE*, apolipoprotein E; RR, relative risk; BBB, blood-based biomarkers; DMT, disease-modifying therapies. The risk operationalization of “low” (RR = 1–2), “moderate” (RR = 2–4), and “high” (RR > 4) intended to be indicative and is used for illustrative purposes only.

**Fig. 1:**
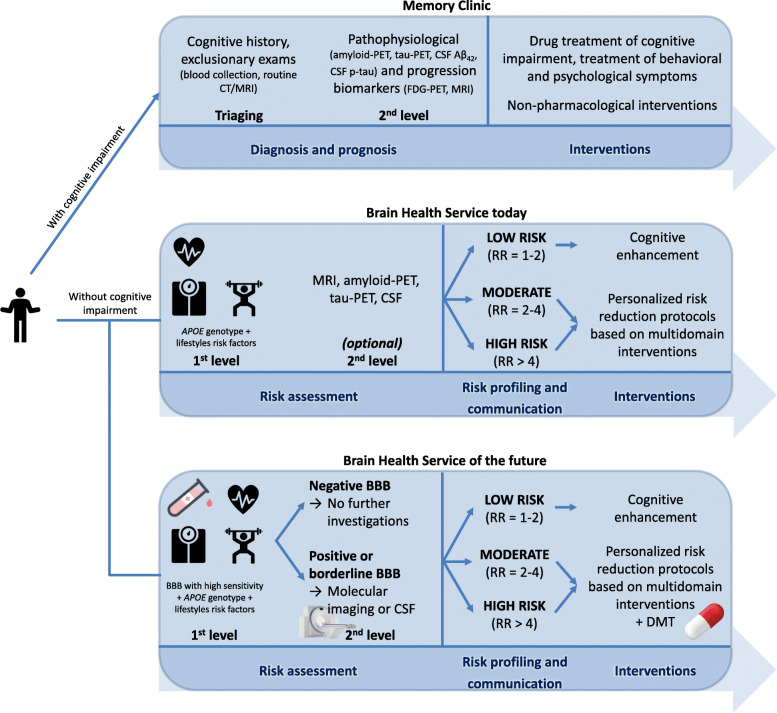
BHS activities today and in the next few years, in comparison with memory clinics

Figure 1 provides an example of how BHSs might operate.

## Conclusion

Despite the many organizational and structural challenges to be faced, we envision that the development of BHSs will play a key role in the fight against the increasing dementia prevalence by embracing the needs of cognitively unimpaired individuals who wish to preserve or improve their cognitive abilities.

## Data Availability

Data sharing is not applicable to this article as no datasets were generated or analyzed during the current study.

## Abbreviations

BHS: Brain Health Services
CAIDE: Cardiovascular Risk Factors, Aging, and Incidence of Dementia
MRI: Magnetic resonance imaging
SCD: Subjective cognitive decline
